# Crystal structure of 4-fluoro-*N*-[2-(4-fluoro­benzo­yl)hydra­zine-1-carbono­thio­yl]benzamide

**DOI:** 10.1107/S1600536814015761

**Published:** 2014-08-01

**Authors:** Syadza Firdausiah, Ameera Aqeela Salleh Huddin, Siti Aishah Hasbullah, Bohari M. Yamin, Siti Fairus M. Yusoff

**Affiliations:** a School of Chemical Sciences and Food Technology, Universiti Kebangsaan Malaysia, 43600 Bangi, Selangor, Malaysia

**Keywords:** crystal structure, hydrogen bonds, semithiocarbazide

## Abstract

In the title compound, C_15_H_11_F_2_N_3_O_2_S, the dihedral angle between the fluoro­benzene rings is 88.43 (10)° and that between the central semithiocarbazide grouping is 47.00 (11)°. The dihedral angle between the amide group and attached fluoro­benzene ring is 50.52 (11)°; the equivalent angle between the carbonyl­thio­amide group and its attached ring is 12.98 (10)°. The major twists in the mol­ecule occur about the C—N—N—C bonds [torsion angle = −138.7 (2)°] and the C_ar_—C_ar_—C—N (ar = aromatic) bonds [−132.0 (2)°]. An intra­molecular N—H⋯O hydrogen bond occurs, which generates an *S*(6) ring. In the crystal, the mol­ecules are linked by N—H⋯O and N—H⋯S hydrogen bonds, generating (001) sheets. Weak C—H⋯O and C—H⋯F inter­actions are also observed.

## Related literature   

For further synthetic details and the crystal structures of related thio­urea derivatives, see: Yamin & Yusof (2003*a* ▶,*b* ▶); Yusof *et al.* (2003 ▶); For a metal complex with a similar ligand, see: Ke *et al.* (2007 ▶).
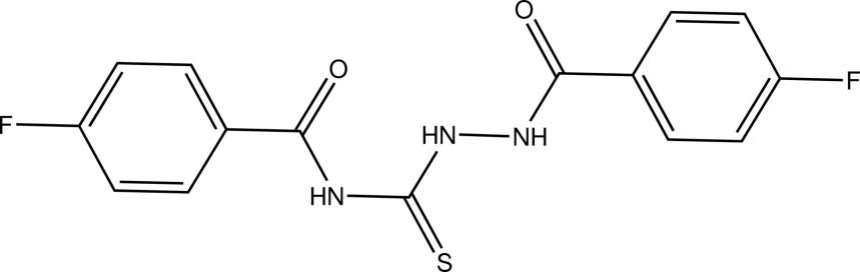


## Experimental   

### Crystal data  

C_15_H_11_F_2_N_3_O_2_S
*M* *_r_* = 335.33
Orthorhombic, 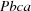

*a* = 11.6172 (6) Å
*b* = 8.4086 (5) Å
*c* = 30.0002 (16) Å
*V* = 2930.6 (3) Å^3^
*Z* = 8
Mo *K*α radiation
μ = 0.26 mm^−1^
*T* = 296 K
0.50 × 0.12 × 0.08 mm

### Data collection  

Bruker SMART APEX CCD diffractometer
Absorption correction: multi-scan (*SADABS*; Bruker, 2000 ▶) *T*_min_ = 0.883, *T*_max_ = 0.980
76830 measured reflections
2886 independent reflections
2024 reflections with *I* > 2σ(*I*)
*R*_int_ = 0.137

### Refinement  

*R*[*F* ^2^ > 2σ(*F* ^2^)] = 0.046
*wR*(*F* ^2^) = 0.099
*S* = 1.04
2886 reflections
208 parameters
H-atom parameters constrained
Δρ_max_ = 0.27 e Å^−3^
Δρ_min_ = −0.21 e Å^−3^

### 

Data collection: *SMART* (Bruker, 2000 ▶); cell refinement: *SAINT* (Bruker, 2000 ▶); data reduction: *SAINT*; program(s) used to solve structure: *SHELXS97* (Sheldrick, 2008 ▶); program(s) used to refine structure: *SHELXL97* (Sheldrick, 2008 ▶); molecular graphics: *SHELXTL* (Sheldrick, 2008 ▶); software used to prepare material for publication: *SHELXTL* and *PLATON* (Spek, 2009 ▶).

## Supplementary Material

Crystal structure: contains datablock(s) global, I. DOI: 10.1107/S1600536814015761/hb7245sup1.cif

Structure factors: contains datablock(s) I. DOI: 10.1107/S1600536814015761/hb7245Isup2.hkl

**Fig. 1.**
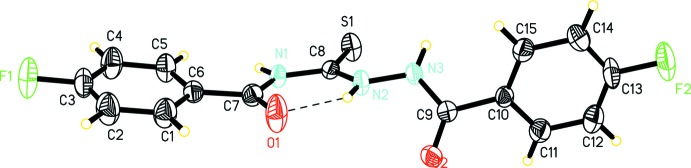
: Molecular structure of (I) with 50% probability displacement ellipsoids. The dashes line indicates the intramolecular hydrogen bond.

. DOI: 10.1107/S1600536814015761/hb7245fig1.tif
Mol­ecular structure of (I) with 50% probability displacement ellipsoids. The dashes line indicates the intra­molecular hydrogen bond.

**Fig. 2.**
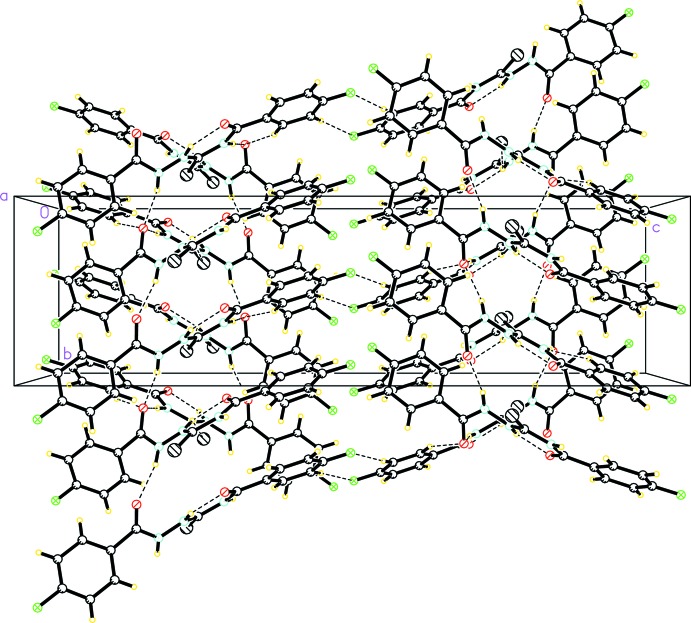
: Unit-cell packing for (I) in the unit cell viewed down a axis. The dashes lines indicate hydrogen bonds.

. DOI: 10.1107/S1600536814015761/hb7245fig2.tif
Unit-cell packing for (I) in the unit cell viewed down a axis. The dashes lines indicate hydrogen bonds.

CCDC reference: 1012471

Additional supporting information:  crystallographic information; 3D view; checkCIF report

## Figures and Tables

**Table 1:** Hydrogen-bond geometry (Å, °)

*D*—H⋯*A*	*D*—H	H⋯*A*	*D*⋯*A*	*D*—H⋯*A*
N2—H2*A*⋯O1	0.86	1.89	2.571 (2)	135
N2—H2*A*⋯S1^i^	0.86	2.84	3.3875 (18)	123
N1—H1*A*⋯O2^ii^	0.86	2.33	3.165 (2)	165
N3—H3⋯O2^iii^	0.86	2.10	2.942 (2)	166
C4—H4⋯F1^iv^	0.93	2.49	3.409 (3)	169
C5—H5⋯O2^ii^	0.93	2.45	3.345 (3)	160

Symmetry codes: (i) 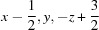; (ii) 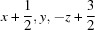; (iii) 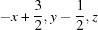; (iv) 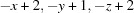.

## supplementary crystallographic information

### S1. Experimental

30 ml acetone containing 4-fluorobenzoyl isothiocyanate (1.81 g, 0.01 mol) was mixed with hydrazine (0.16 g, 0.005 mol). The mixture was refluxed for 2 hours. The solution was filtered and left to evaporate at room temperature. The white precipitate obtained after a few days, was washed with water and cold ethanol. Colourless blocks of the title compound were obtained by recrystallization from ethanol solution.

### S2. Refinement

After location in the difference map, the H-atoms attached to the C and N atoms were fixed geometrically at ideal positions and allowed to ride on the parent atoms with C—H = 0.93-0.97 Å, N—H = 0.86 Å and with *U*_iso_(H)=1.2*U*_eq_(C or N).

### Crystal data

**Table:**

C_15_H_11_F_2_N_3_O_2_S	*Z* = 8
*M**_r_* = 335.33	*F*(000) = 1376
Orthorhombic, *P**b**c**a*	*D*_x_ = 1.520 Mg m^−^^3^
Hall symbol: -P 2ac 2ab	Mo *K*α radiation, λ = 0.71073 Å
*a* = 11.6172 (6) Å	µ = 0.26 mm^−^^1^
*b* = 8.4086 (5) Å	*T* = 296 K
*c* = 30.0002 (16) Å	Block, colorless
*V* = 2930.6 (3) Å^3^	0.50 × 0.12 × 0.08 mm

### Data collection

**Table:**

Bruker SMART APEX CCD diffractometer	2886 independent reflections
Radiation source: fine-focus sealed tube	2024 reflections with *I* > 2σ(*I*)
Graphite monochromator	*R*_int_ = 0.137
ω scans	θ_max_ = 26.0°, θ_min_ = 3.1°
Absorption correction: multi-scan (*SADABS*; Bruker, 2000)	*h* = −14→14
*T*_min_ = 0.883, *T*_max_ = 0.980	*k* = −10→10
76830 measured reflections	*l* = −37→37

### Refinement

**Table:**

Refinement on *F*^2^	Primary atom site location: structure-invariant direct methods
Least-squares matrix: full	Secondary atom site location: difference Fourier map
*R*[*F*^2^ > 2σ(*F*^2^)] = 0.046	Hydrogen site location: inferred from neighbouring sites
*wR*(*F*^2^) = 0.099	H-atom parameters constrained
*S* = 1.04	*w* = 1/[σ^2^(*F*_o_^2^) + (0.0354*P*)^2^ + 2.0394*P*] where *P* = (*F*_o_^2^ + 2*F*_c_^2^)/3
2886 reflections	(Δ/σ)_max_ = 0.001
208 parameters	Δρ_max_ = 0.27 e Å^−^^3^
0 restraints	Δρ_min_ = −0.21 e Å^−^^3^

### Special details

**Table:**

Geometry. All esds (except the esd in the dihedral angle between two l.s. planes) are estimated using the full covariance matrix. The cell esds are taken into account individually in the estimation of esds in distances, angles and torsion angles; correlations between esds in cell parameters are only used when they are defined by crystal symmetry. An approximate (isotropic) treatment of cell esds is used for estimating esds involving l.s. planes.
Refinement. Refinement of F^2^ against ALL reflections. The weighted R-factor wR and goodness of fit S are based on F^2^, conventional R-factors R are based on F, with F set to zero for negative F^2^. The threshold expression of F^2^ > 2sigma(F^2^) is used only for calculating R-factors(gt) etc. and is not relevant to the choice of reflections for refinement. R-factors based on F^2^ are statistically about twice as large as those based on F, and R- factors based on ALL data will be even larger.

### Fractional atomic coordinates and isotropic or equivalent isotropic displacement parameters (Å^2^)

**Table:**

	*x*	*y*	*z*	*U*_iso_*/*U*_eq_	
S1	0.98887 (5)	0.15863 (8)	0.73680 (2)	0.04054 (19)	
O1	0.69608 (15)	0.4074 (2)	0.80598 (6)	0.0585 (6)	
O2	0.64343 (13)	0.34844 (18)	0.67414 (5)	0.0328 (4)	
N1	0.88035 (15)	0.3239 (2)	0.79937 (6)	0.0283 (4)	
H1A	0.9472	0.3290	0.8116	0.034*	
N2	0.76928 (15)	0.2357 (2)	0.74100 (6)	0.0308 (5)	
H2A	0.7137	0.2871	0.7533	0.037*	
N3	0.74823 (15)	0.1473 (2)	0.70307 (5)	0.0278 (4)	
H3	0.7784	0.0548	0.6995	0.033*	
C1	0.7245 (2)	0.5302 (3)	0.88928 (8)	0.0444 (7)	
H1	0.6558	0.5469	0.8741	0.053*	
C2	0.7347 (2)	0.5802 (3)	0.93299 (8)	0.0511 (7)	
H2	0.6738	0.6303	0.9474	0.061*	
C3	0.8362 (2)	0.5540 (3)	0.95425 (8)	0.0430 (7)	
C4	0.9276 (2)	0.4826 (3)	0.93441 (8)	0.0479 (7)	
H4	0.9959	0.4667	0.9500	0.057*	
C5	0.9171 (2)	0.4339 (3)	0.89050 (8)	0.0402 (6)	
H5	0.9792	0.3861	0.8762	0.048*	
C6	0.81520 (19)	0.4557 (3)	0.86779 (7)	0.0300 (5)	
C7	0.79209 (19)	0.3964 (3)	0.82191 (7)	0.0315 (5)	
C8	0.87407 (18)	0.2428 (3)	0.75895 (7)	0.0258 (5)	
C9	0.67935 (17)	0.2104 (3)	0.67201 (7)	0.0254 (5)	
C10	0.64585 (18)	0.1018 (3)	0.63505 (7)	0.0263 (5)	
C11	0.6553 (2)	0.1549 (3)	0.59144 (7)	0.0383 (6)	
H11	0.6838	0.2563	0.5858	0.046*	
C12	0.6228 (2)	0.0589 (3)	0.55647 (8)	0.0481 (7)	
H12	0.6300	0.0931	0.5271	0.058*	
C13	0.5797 (2)	−0.0878 (3)	0.56616 (9)	0.0503 (7)	
C15	0.6004 (2)	−0.0473 (3)	0.64350 (8)	0.0360 (6)	
H15	0.5929	−0.0828	0.6727	0.043*	
F1	0.84583 (15)	0.6012 (2)	0.99733 (5)	0.0681 (5)	
F2	0.54797 (19)	−0.1828 (2)	0.53146 (6)	0.0870 (7)	
C14	0.5660 (2)	−0.1434 (3)	0.60860 (9)	0.0491 (7)	
H14	0.5344	−0.2433	0.6139	0.059*	

### Atomic displacement parameters (Å^2^)

**Table:**

	*U*^11^	*U*^22^	*U*^33^	*U*^12^	*U*^13^	*U*^23^
S1	0.0271 (3)	0.0533 (4)	0.0412 (4)	0.0032 (3)	−0.0018 (3)	−0.0162 (3)
O1	0.0387 (11)	0.0887 (16)	0.0482 (11)	0.0263 (10)	−0.0169 (9)	−0.0332 (11)
O2	0.0344 (9)	0.0280 (9)	0.0359 (9)	0.0044 (8)	−0.0042 (7)	−0.0043 (7)
N1	0.0248 (9)	0.0357 (11)	0.0242 (9)	−0.0003 (8)	−0.0042 (8)	−0.0055 (8)
N2	0.0269 (10)	0.0392 (12)	0.0262 (10)	0.0057 (9)	−0.0042 (8)	−0.0118 (9)
N3	0.0280 (9)	0.0286 (10)	0.0270 (10)	0.0032 (9)	−0.0059 (8)	−0.0073 (8)
C1	0.0390 (15)	0.0567 (18)	0.0374 (14)	0.0123 (13)	−0.0061 (12)	−0.0095 (13)
C2	0.0505 (17)	0.0635 (19)	0.0394 (15)	0.0131 (15)	0.0054 (13)	−0.0154 (14)
C3	0.0531 (17)	0.0494 (17)	0.0265 (13)	−0.0062 (14)	−0.0001 (12)	−0.0112 (12)
C4	0.0389 (14)	0.067 (2)	0.0377 (15)	0.0004 (14)	−0.0096 (12)	−0.0118 (14)
C5	0.0347 (14)	0.0535 (17)	0.0323 (13)	0.0045 (12)	−0.0021 (11)	−0.0116 (12)
C6	0.0316 (13)	0.0303 (13)	0.0280 (12)	−0.0002 (10)	−0.0017 (10)	−0.0027 (11)
C7	0.0300 (12)	0.0332 (14)	0.0314 (13)	0.0042 (10)	−0.0046 (10)	−0.0026 (11)
C8	0.0294 (12)	0.0253 (12)	0.0228 (11)	−0.0025 (10)	−0.0034 (10)	0.0009 (9)
C9	0.0214 (11)	0.0280 (13)	0.0267 (12)	−0.0014 (10)	0.0019 (9)	−0.0010 (10)
C10	0.0233 (11)	0.0289 (13)	0.0267 (12)	0.0027 (10)	−0.0056 (9)	−0.0036 (10)
C11	0.0464 (14)	0.0374 (15)	0.0313 (13)	−0.0068 (13)	−0.0024 (11)	0.0013 (12)
C12	0.0634 (18)	0.0546 (18)	0.0261 (13)	−0.0020 (15)	−0.0060 (12)	−0.0041 (13)
C13	0.0603 (18)	0.0505 (18)	0.0399 (16)	−0.0067 (15)	−0.0182 (13)	−0.0189 (14)
C15	0.0390 (14)	0.0368 (15)	0.0321 (13)	−0.0051 (12)	−0.0082 (11)	0.0020 (11)
F1	0.0741 (11)	0.0980 (14)	0.0322 (8)	−0.0006 (10)	−0.0036 (8)	−0.0258 (9)
F2	0.1324 (18)	0.0781 (14)	0.0506 (11)	−0.0280 (12)	−0.0284 (11)	−0.0258 (10)
C14	0.0589 (18)	0.0370 (16)	0.0514 (17)	−0.0158 (14)	−0.0154 (14)	−0.0037 (13)

### Geometric parameters (Å, º)

**Table:**

S1—C8	1.650 (2)	C4—C5	1.385 (3)
O1—C7	1.217 (3)	C4—H4	0.9300
O2—C9	1.235 (3)	C5—C6	1.378 (3)
N1—C7	1.371 (3)	C5—H5	0.9300
N1—C8	1.393 (3)	C6—C7	1.488 (3)
N1—H1A	0.8600	C9—C10	1.488 (3)
N2—C8	1.333 (3)	C10—C15	1.384 (3)
N2—N3	1.381 (2)	C10—C11	1.387 (3)
N2—H2A	0.8600	C11—C12	1.377 (3)
N3—C9	1.338 (3)	C11—H11	0.9300
N3—H3	0.8600	C12—C13	1.362 (4)
C1—C2	1.382 (3)	C12—H12	0.9300
C1—C6	1.385 (3)	C13—F2	1.363 (3)
C1—H1	0.9300	C13—C14	1.366 (4)
C2—C3	1.359 (4)	C15—C14	1.381 (3)
C2—H2	0.9300	C15—H15	0.9300
C3—F1	1.357 (3)	C14—H14	0.9300
C3—C4	1.358 (4)		
			
C7—N1—C8	127.43 (18)	O1—C7—N1	121.7 (2)
C7—N1—H1A	116.3	O1—C7—C6	120.2 (2)
C8—N1—H1A	116.3	N1—C7—C6	118.04 (19)
C8—N2—N3	121.27 (18)	N2—C8—N1	114.95 (18)
C8—N2—H2A	119.4	N2—C8—S1	123.80 (16)
N3—N2—H2A	119.4	N1—C8—S1	121.23 (15)
C9—N3—N2	117.80 (18)	O2—C9—N3	122.6 (2)
C9—N3—H3	121.1	O2—C9—C10	121.8 (2)
N2—N3—H3	121.1	N3—C9—C10	115.58 (19)
C2—C1—C6	121.0 (2)	C15—C10—C11	119.7 (2)
C2—C1—H1	119.5	C15—C10—C9	121.3 (2)
C6—C1—H1	119.5	C11—C10—C9	119.0 (2)
C3—C2—C1	118.0 (2)	C12—C11—C10	120.5 (2)
C3—C2—H2	121.0	C12—C11—H11	119.7
C1—C2—H2	121.0	C10—C11—H11	119.7
F1—C3—C4	118.8 (2)	C13—C12—C11	118.0 (2)
F1—C3—C2	118.1 (2)	C13—C12—H12	121.0
C4—C3—C2	123.0 (2)	C11—C12—H12	121.0
C3—C4—C5	118.6 (2)	C12—C13—F2	117.8 (2)
C3—C4—H4	120.7	C12—C13—C14	123.5 (2)
C5—C4—H4	120.7	F2—C13—C14	118.7 (3)
C6—C5—C4	120.5 (2)	C14—C15—C10	120.1 (2)
C6—C5—H5	119.8	C14—C15—H15	119.9
C4—C5—H5	119.8	C10—C15—H15	119.9
C5—C6—C1	118.9 (2)	C13—C14—C15	118.2 (3)
C5—C6—C7	124.6 (2)	C13—C14—H14	120.9
C1—C6—C7	116.4 (2)	C15—C14—H14	120.9
			
C8—N2—N3—C9	−138.7 (2)	C7—N1—C8—N2	−0.5 (3)
C6—C1—C2—C3	0.0 (4)	C7—N1—C8—S1	−178.67 (18)
C1—C2—C3—F1	−179.2 (3)	N2—N3—C9—O2	6.9 (3)
C1—C2—C3—C4	0.5 (5)	N2—N3—C9—C10	−171.47 (17)
F1—C3—C4—C5	179.6 (3)	O2—C9—C10—C15	−127.2 (2)
C2—C3—C4—C5	−0.1 (4)	N3—C9—C10—C15	51.2 (3)
C3—C4—C5—C6	−0.9 (4)	O2—C9—C10—C11	49.5 (3)
C4—C5—C6—C1	1.4 (4)	N3—C9—C10—C11	−132.0 (2)
C4—C5—C6—C7	−175.0 (2)	C15—C10—C11—C12	−1.9 (4)
C2—C1—C6—C5	−1.0 (4)	C9—C10—C11—C12	−178.6 (2)
C2—C1—C6—C7	175.7 (3)	C10—C11—C12—C13	1.1 (4)
C8—N1—C7—O1	−6.0 (4)	C11—C12—C13—F2	−179.8 (3)
C8—N1—C7—C6	171.2 (2)	C11—C12—C13—C14	0.7 (4)
C5—C6—C7—O1	172.8 (3)	C11—C10—C15—C14	0.9 (4)
C1—C6—C7—O1	−3.6 (4)	C9—C10—C15—C14	177.6 (2)
C5—C6—C7—N1	−4.4 (4)	C12—C13—C14—C15	−1.7 (4)
C1—C6—C7—N1	179.1 (2)	F2—C13—C14—C15	178.8 (3)
N3—N2—C8—N1	−174.21 (18)	C10—C15—C14—C13	0.8 (4)
N3—N2—C8—S1	3.9 (3)		

### Hydrogen-bond geometry (Å, º)

**Table:**

*D*—H···*A*	*D*—H	H···*A*	*D*···*A*	*D*—H···*A*
N2—H2*A*···O1	0.86	1.89	2.571 (2)	135
N2—H2*A*···S1^i^	0.86	2.84	3.3875 (18)	123
N1—H1*A*···O2^ii^	0.86	2.33	3.165 (2)	165
N3—H3···O2^iii^	0.86	2.10	2.942 (2)	166
C4—H4···F1^iv^	0.93	2.49	3.409 (3)	169
C5—H5···O2^ii^	0.93	2.45	3.345 (3)	160

Symmetry codes: (i) *x*−1/2, *y*, −*z*+3/2; (ii) *x*+1/2, *y*, −*z*+3/2; (iii) −*x*+3/2, *y*−1/2, *z*; (iv) −*x*+2, −*y*+1, −*z*+2.
