# Supplementary figures and images for: Crystal structure of 4-fluoro-N-[2-(4-fluoro­benzo­yl)hydra­zine-1-carbono­thio­yl]benzamide
Source: Acta Crystallogr Sect E Struct Rep Online. 2014 Aug 1;70(Pt 9):o915–6. doi: 10.1107/S1600536814015761 (PMC4186173; doi:10.1107/S1600536814015761)

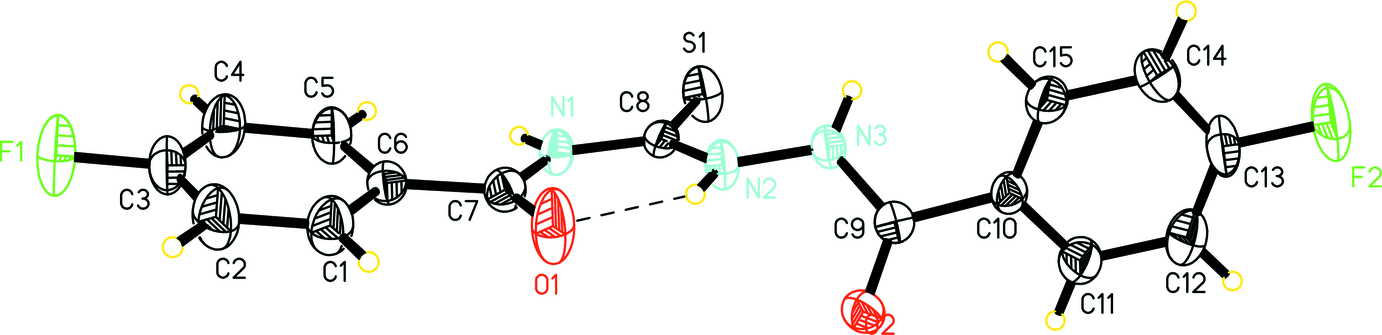

Supplement: Supplementary file 3 [file e-70-0o915-fig1.tif]

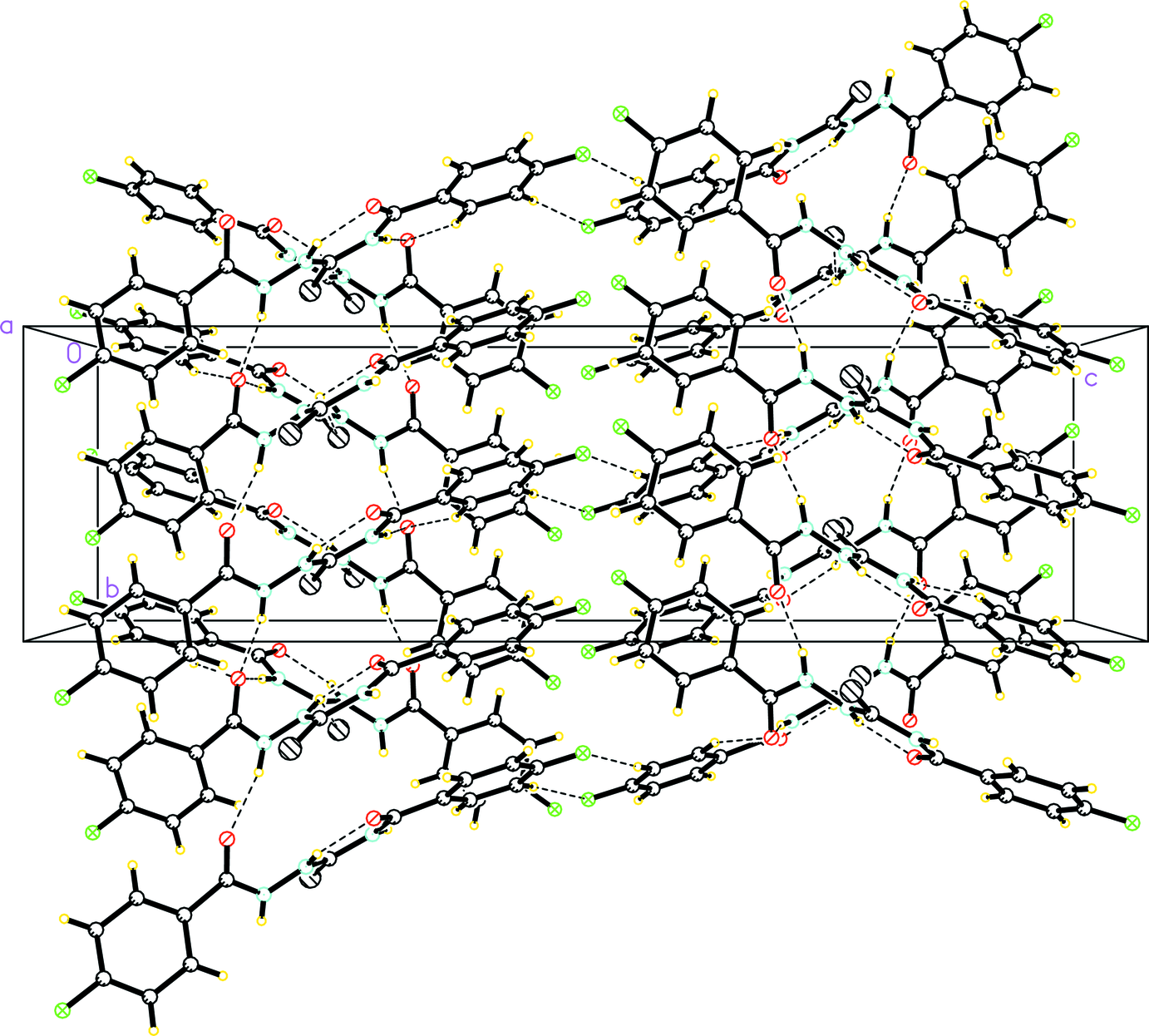

Supplement: Supplementary file 4 [file e-70-0o915-fig2.tif]
